# Resource Planning for Neglected Tropical Disease (NTD) Control Programs: Feasibility Study of the Tool for Integrated Planning and Costing (TIPAC)

**DOI:** 10.1371/journal.pntd.0002619

**Published:** 2014-02-27

**Authors:** Olivier J. Wouters, Philip W. Downs, Kathryn L. Zoerhoff, Kathryn R. Crowley, Hannah Frawley, Jennifer Einberg, Brian K. Chu, Molly A. Brady, Roland Oscar, Mireille Jeudi, Anne-Marie Desormeaux, Karleen Coly, Abdel N. Direny, Garib D. Thakur, Raj K. Pokharel, Shekhar Sharma, Dharmpal P. Raman, Santigie Sesay, Mustapha Sonnie, Bernard Kilembe, Upendo Mwingira, Aya Yajima

**Affiliations:** 1 RTI International, Washington, D.C., United States of America; 2 LSE Health, London School of Economics and Political Science, London, United Kingdom; 3 Iota Ink LLC, Seattle, Washington, United States of America; 4 The Task Force for Global Health, Decatur, Georgia, United States of America; 5 Ministry of Public Health and Population, Port au Prince, Haiti; 6 IMA World Health, New Windsor, Maryland, United States of America; 7 IMA World Health, Port au Prince, Haiti; 8 Ministry of Health and Population, Kathmandu, Nepal; 9 National Trachoma Program, Kathmandu, Nepal; 10 RTI International, Kathmandu, Nepal; 11 Ministry of Health and Sanitation, Freetown, Sierra Leone; 12 Helen Keller International, Freetown, Sierra Leone; 13 Ministry of Health and Social Welfare, Dar es Salaam, Tanzania; 14 Department of Control of Neglected Tropical Diseases, World Health Organization, Geneva, Switzerland; Georgetown University, United States of America

## Introduction

Neglected tropical diseases (NTDs) cause significant morbidity and mortality worldwide and impose a large economic burden on endemic countries [1]. In 2006, the United States Agency for International Development (USAID) founded the NTD Control Program to target five NTDs in African, Asian, and Latin American countries, namely, lymphatic filariasis (LF), onchocerciasis, schistosomiasis, soil-transmitted helminthiases (STH), and trachoma; the three targeted STH infections are ascariasis, hookworm, and trichuriasis. The NTD Control Program supported national NTD control and elimination programs' efforts to integrate and scale up delivery of preventive chemotherapy (PC) [2]. PC is the administration of safe, single-dose drugs, either alone or in combination, as a public health intervention against targeted NTDs. Administration is characterized by population-based diagnosis,population-based treatment,and implementation at regular intervals. PC can be delivered as universal chemotherapy (i.e., mass drug administration [MDA]), where the entire population of an area is targeted; targeted chemotherapy, where only high-risk groups (e.g., school age children) are targeted; or selective chemotherapy, where only screened individuals found or suspected to be infected are targeted [3]. Between October 2006 and March 2012, the program provided 589 million NTD treatments through the collaborative efforts of ministries of health, implementing partners, funders, and pharmaceutical donation programs.

The implementation of integrated NTD programs at the full national scale remains an important objective in many endemic countries [4]–[8]. Several theoretical frameworks for integration have been proposed; most protocols stress the importance of long-term commitments and concerted efforts of partnerships to realize NTD control and elimination objectives [9]–[14]. However, there is currently a paucity of economic evidence on the costs of integrated PC delivery for NTDs, primarily due to the significant variation in program structures and operations [14]. Given the scarce resources and substantial costs associated with NTD control and elimination, there is therefore a need to accurately determine the cost of program implementation. It is also important to delineate funding commitments to ensure that additional assistance is used to complement available resources, rather than duplicate or replace previous efforts.

To allow governments to more easily enumerate costs and funding commitments for NTD control and elimination, the NTD Control Program developed the Tool for Integrated Planning and Costing (TIPAC). The TIPAC, a versatile planning and costing instrument, is designed to be used by members of a NTD program at the national level. For countries with decentralized political structures, the TIPAC can also be implemented at a subnational administrative level. NTD program and financial managers are the primary users of the tool; the involvement of other personnel, including representatives from partner organizations and ministries of education, improves the accuracy and completeness of the TIPAC data.

The TIPAC implementation process includes four main phases:

1. Background data collection: country coordinators compile, review, and verify demographic, epidemiologic, and cost classification data. In countries where individual disease control programs are not integrated, this phase may generate fruitful discussions among stakeholders and stimulate collaboration on program planning, outreach, implementation, and monitoring.
2. Data entry: a focal person appointed by the national program enters the planned activity costs. The national strategic plans for NTD control and elimination, also referred to as master plans, serve as the guiding documents for data entry. As the tool is populated, integration opportunities and areas of overlap and duplication are identified. After the costs are entered, stakeholder meetings are convened to identify drug and funding commitments.
3. Finalization and approval: the entered data is reviewed by all stakeholders and approved for use by ministry of health representatives from the national NTD control and elimination program.
4. Results application: the results can be used to inform and guide annual work plans, drug applications, donor coordination efforts, and advocacy and fundraising strategies. The TIPAC is able to convert the information in the tool for use during another funding year, thereby facilitating data entry in subsequent years.

The aim of this feasibility study is to assess whether the TIPAC effectively informs and facilitates country program decision-making and the integration of program activities. This study presents excerpts from data collected in two African countries (Sierra Leone, fiscal year [FY] Oct. 2010–Sept. 2011, and Tanzania, FY Oct. 2010–Sept. 2011), one Asian country (Nepal, FY Jul. 2010–Jul. 2011), and one Latin American country (Haiti, FY Oct. 2011–Sept. 2012). The lessons learned from implementing the TIPAC in these four countries can guide the planning and costing of annual NTD control and elimination activities in other NTD-endemic countries.

## Methods

### The TIPAC structure

The initial 2009 tool was a one-page spreadsheet in Microsoft Excel which allowed users to enter cost and funding levels for planned NTD program activities. A subsequent multisheet Excel workbook was developed, which enabled users to more accurately specify activity costs (i.e., list the component costs of individual activities). In 2009 and 2010, the tool was implemented in 14 countries (Burkina Faso, Cameroon, Ghana, Haiti, Nepal, Niger, the Philippines, Senegal, Sierra Leone, South Sudan, Tanzania, Togo, Uganda, and Vietnam) by technical advisers in collaboration with ministries of health. Based on these experiences, it was revised in 2012 to strengthen its functionality and user-friendliness, increase the transparency of cost inputs, and enable more advanced customizable reports. Prior to 2012, the tool was known as the Funding Gap Analysis Tool (FGAT). The newest version of the tool, available in Bahasa Indonesia, English, French, Portuguese, and Spanish, facilitates program planning and budgeting. The main improvements include a timeline of activities, an expanded capacity to enter information for non-PC NTDs, and a five-year projection of activity costs and PC drug needs.

The current TIPAC version was developed in Microsoft Excel 2007. The tool applies an iterative approach to guide users through data entry, with user forms designed in Visual Basic for Applications (VBA). It includes four data entry modules and one reports module:

1. Base data: stores demographic, epidemiologic, and program information for use throughout the tool.
2. Activity costs: collects costs of NTD program activities.
3. PC drug acquisition: estimates PC drug demand and costs (see Text S1 for the PC target population and drug demand algorithms).
4. Funders: saves information about government and donor budgets.
5. Reports: synthesizes results and facilitates data analysis; the tool automatically generates summary analyses (i.e., figures and tables).

The planning and costing of activities are based on World Health Organization (WHO) treatment guidelines for implementing integrated PC disease control programs and the scope of the national strategic plans [15]–[19]. Table S1 summarizes the default activities and sub-activities captured by the tool; users are also able to add other activities and sub-activities.

### Assessment of feasibility

This paper examines the feasibility of the TIPAC to support country program decision making and the integration of program activities. Feasibility is assessed based on the five tool objectives:

1. Estimate the costs of implementing integrated NTD programs in accordance with international guidelines and national plans of action.
2. Quantify the existing resources of governments and donors.
3. Identify the funding gaps of the national NTD programs.
4. Encourage the rational allocation of resources and coordination between governments, implementing partners, and donors.
5. Facilitate the identification of integration opportunities and annual planning of NTD programs.

The countries represented in this study (Haiti, Nepal, Sierra Leone, and Tanzania) were selected to provide a diverse geographic distribution and varied disease landscapes for analysis. Of the four, Tanzania is the only country treating all five diseases, while Haiti, Nepal, and Sierra Leone treat two, three, and four diseases, respectively. Haiti and Sierra Leone targeted 100% of the endemic districts for treatment. Nepal targeted all districts for STH treatment, but is continuing to scale up treatment for LF and trachoma. Tanzania only provided data for 25 districts that were targeted with USAID funding, or 19% of the total number of districts. An additional 36 districts in Tanzania were supported by the African Programme for Onchocerciasis Control (APOC), but were not included in the TIPAC. Tanzania is using a phased approach to scale up NTD treatment and has not yet reached 100% geographic coverage. Key information on the national NTD programs is summarized in Table 1.

**Table 1:** Overview of NTD control programs.*

Country	LF	Oncho	SCH	STH	Trachoma	Total population	# of people targeted	Total # of districts	# of districts targeted
Haiti	✓			✓		9,897,749	9,254,397	140	140
Nepal	✓			✓	✓	28,076,055	17,151,069	75	75
Sierra Leone	✓	✓	✓	✓		5,890,280	5,005,105	14	14
Tanzania	✓	✓	✓	✓	✓	41,422,687	7,504,657	130	25

* The demographic and district figures from Table 1 represent country situations at the time of data entry.

All monetary values entered into the TIPAC were converted to US dollars ($) using the exchange rate provided by a government employee or technical adviser at the time of data entry (i.e., $1 was equivalent to HTG 40.0 [Haiti], NRS 75.7 [Nepal], SLL 3,920.0 [Sierra Leone], and TZS 1,550.0 [Tanzania]). All the data were initially entered into an earlier version of the TIPAC and later transferred to an updated version. The cost and funding data were obtained from the perspective of national NTD programs.

## Results

The level of time and effort required for data entry varied across the four countries depending on the size, scope, complexity, and maturity of the programs. In most cases, ten working days were needed to enter the vast majority of the data; additional input and refinement was possible as new information became available. Each country program received in-country assistance from a trained facilitator who introduced the TIPAC to program managers and provided technical support during implementation. Programs were encouraged to appoint a national focal person to maintain and update the TIPAC for subsequent work planning. In all four countries, data were successfully generated that contributed to the following key objectives.

### Objective 1: Estimate the costs of implementing integrated NTD programs in accordance with international guidelines and national plans of action

The epidemiological data captured in the Base Data module were used by countries to set targets and estimate annual costs to operationalize national plans of action. Table 2 summarizes the target age group, target population, and number of districts targeted for each disease in Sierra Leone. All four countries inputted program costs for PC and non-PC activities and complementary NTD control strategies (e.g., vector control). Both economic and financial costs were generated by the tool; financial costs excluded the monetary value of donated drugs (i.e., only cash disbursements were considered). Table 3 lists Haitian activity costs captured in the tool, excluding salaries and drug costs. Reports generated by the TIPAC also showed the cost distribution by sub-activity or line item.

**Table 2:** Sierra Leone base data (FY Oct. 2010–Sept. 2011).

Disease	Target age group for PC	Target population for PC	Number of districts targeted for PC
LF	≥5 years	4,888,932	14
Oncho (Round 1)	≥5 years	2,403,894	12
Oncho (Round 2)	≥5 years	0	0
SCH	School age	485,739	7
	High-risk adults	1,303,644	7
STH (Round 1)	Preschool age	0	0
	School age	1,590,376	14
	High-risk adults	3,298,556	14
STH (Round 2)	School age	485,739	7
Trachoma	<6 months	0	0
	6–59 months	0	0
	≥5 years	0	0

**Table 3:** Haiti activity costs (in $, FY Oct. 2011–Sept. 2012).

Activity	Total cost	Percent
**Implementation costs**		
PC drug distribution	1,255,079	45.1%
Social mobilization	755,125	27.1%
PC training	473,902	17.0%
Medication for side effects	145,913	5.2%
Monitoring and evaluation	56,799	2.0%
PC preparation	46,270	1.7%
Strategic planning	14,965	0.5%
Drug logistics	14,590	0.5%
Department supervision	8,759	0.3%
**Operational costs**		
Office equipment	14,075	0.5%
**Total program costs**	**2,785,477**	**100.0%**

In addition, countries were able to calculate the average projected cost per person for PC NTDs. Table 4 details the estimated aggregate and per-person economic costs in Haiti. These included implementation and operational costs, as well as the costs of donated and purchased drugs; salaries were excluded from these estimates. The NTD program managers were then able to determine the relative cost differences between diseases, which allowed for more informed planning and opportunities to reduce costs in subsequent years.

**Table 4:** Haiti economic cost per person targeted (in $, FY Oct. 2011–Sept. 2012).

	LF	Oncho	SCH	STH	Trachoma	All PC
Total program costs	1,958,292	0	0	1,533,513	0	3,491,805
Number of persons targeted	9,254,397	0	0	2,474,438	0	9,254,397
Cost per person targeted	0.21	0.00	0.00	0.62	0.00	0.38
Number of districts targeted	140	0	0	140	0	140

### Objective 2: Quantify the existing resources of governments and donors

To illustrate the ability to quantify existing resources from government and donor budgets (e.g., bilateral donors and nongovernmental organizations [NGOs]), Table 5 shows the overall funding by activity and funding source in Nepal; the table includes salaries, but excludes drug costs and donations. All funds in Nepal were assigned to specific activities, sub-activities, and districts, to match the funder's intent. The TIPAC results reflected the government of Nepal's funding commitment, as well as the allocation of resources through a pooled fund. While the money included in the pooled fund was provided by external donors, the government of Nepal decided how to allocate the money. The government funding and the pooled fund accounted for over 70% of the total funding.

**Table 5:** Nepal funders, by activity (in $, FY Jul. 2010–Jul. 2011).*

Activity	Cost	Funding	Gap	Government	Pool fund	USAID	CNTD/LSTM	Embassy of India	Lions Club Int'l
**Implementation costs**									
Salaries	986,036	986,036	0	986,036	0	0	0	0	0
Drug logistics	190,372	183,340	7,031	151,192	25,007	7,142	0	0	0
Mapping	28,503	28,503	0	0	0	28,503	0	0	0
PC drug distribution	956,597	934,453	22,145	466,076	324,347	144,029	0	0	0
PC registration	554,030	483,425	70,605	0	0	483,425	0	0	0
PC training	1,308,672	973,329	335,344	68,854	738,171	166,304	0	0	0
Monitoring and evaluation	180,139	151,158	28,981	4,262	59,670	53,293	22,655	0	0
Morbidity control and surgery	137,446	121,181	16,265	0	2,642	3,196	0	29,062	85,687
Social mobilization	492,435	481,316	11,118	230,334	119,551	92,871	38,560	0	0
Strategic planning	139,706	116,113	23,594	89,053	0	16,227	10,834	0	0
**Operational costs**									
Running costs	37,707	37,707	0	20,876	0	16,831	0	0	0
**Total program costs**	**5,011,643**	**4,496,561**	**515,082**	**2,016,682**	**1,269,388**	**1,011,821**	**72,049**	**29,062**	**85,687**

* CNTD = Centre for Neglected Tropical Diseases; LSTM = Liverpool School of Tropical Medicine; USAID = United States Agency for International Development.

In Sierra Leone, staff salaries contributed to 15% of the total program funding, while government resources allocated through the national budgeting process accounted for less than 1% of the total. This data helped the Ministry of Health and Sanitation (MOHS) better understand the annual costs of implementing NTD activities, the financial commitments of donors, and the resources required for scaling up to reach national coverage. It motivated the MOHS to increase their financial commitment for drug distribution, drug storage, and personnel salaries.

### Objective 3: Identify the funding gaps of the national NTD programs

The TIPAC provided program managers with a valuable advocacy tool and an opportunity to communicate to funders how available resources aligned with planned activities and where additional assistance was needed. For example, in the four countries described in this study, the results of the TIPAC were used during annual planning to identify funding gaps and to guide the budget requests for USAID support. Notably, the implementation of the TIPAC in Sierra Leone reaffirmed that morbidity control and surgery (i.e., hydrocele surgery and lymphedema management) was underfunded. Johnson & Johnson therefore recommitted funding for hydrocele surgeries and physician training on novel hydrocele surgery techniques. Thus far, more than 150 patients have received free operations and 50 physicians have obtained supplementary training. In Nepal, the data entry process enabled the program to identify and quantify the funding gap, which corresponded to 10.3% (515,082/5,011,643) of the total budgeted program costs.

Potential gaps in the quantity of drug units required for each targeted PC NTD and the associated costs were automatically calculated for each country based on their respective epidemiologic statuses and the entered program goals for the time period (i.e., population and number of districts targeted for treatment). This information was used to check against drug procurement records to confirm whether quantities of donated and procured drugs were sufficient to cover targeted districts. Table 6 summarizes the drug acquisition data from Tanzania.

**Table 6:** Tanzania drug acquisition (in drug units,* FY Oct. 2010–Sept. 2011).**

Drug	Manufacturer	Donor/funder	Total needed	Total stock	Total funded	Gap
IVM tabs	Merck	Merck & Co.	17,905,801	0	17,905,801	0
ALB tabs (with IVM)	GSK/other	GSK	7,034,422	0	7,034,422	0
PZQ tabs	Multiple	USAID	3,179,394	0	1,041,063	2,138,331
TEO tubes	Multiple		220,817	0	0	220,817
ZMAX POS bottles	Pfizer	ITI	328,104	0	328,104	0
ZMAX tabs	Pfizer	ITI	13,331,922	0	13,331,922	0

* The tool also allowed users to display the monetary value of medicines, instead of the drug units.

** ALB = albendazole; GSK = GlaxoSmithKline; ITI = International Trachoma Initiative; IVM = ivermectin; POS = pediatric oral suspension; PZQ = praziquantel; TEO = tetracycline eye ointment; USAID = United States Agency for International Development; ZMAX = zithromax.

### Objective 4: Encourage the rational allocation of resources and coordination between governments, implementing partners, and donors

In Haiti, populating the TIPAC allowed the various partners, including departments within the Ministry of Public Health and Population (MSPP), to share information on the NTD activities they were conducting or planning. These activities included morbidity management, vector control through the use of long-lasting insecticide treated nets (LLITNs), and a new proposal to implement a second round of mebendazole for STH in targeted districts. Although the second round of mebendazole was not approved by the MSPP, further discussions led the Inter-American Development Bank (IDB) to instead finance a second round of albendazole in five districts. Through this process, the MSPP gained an enhanced understanding of the contributions of its partners and adjusted implementation strategies when appropriate.

Table 7 provides a summary of the work plan matrix and timeline in Haiti generated for the period of October 2011 to September 2012. Based on the user's selection of targeted months and years, the matrix provided a one-year overview of Haiti's activities and subactivities. This output was used by program managers to coordinate among partners and identify any spatial and temporal overlap.

**Table 7:** Haiti annual work plan matrix and timeline (FY Oct. 2011–Sept. 2012).*

	Timeline for implementation											
Activities and Sub-activities	Oct 11	Nov 11	Dec 11	Jan 12	Feb 12	Mar 12	Apr 12	May 12	Jun 12	Jul 12	Aug 12	Sep 12
**Strategic planning**												
National stakeholders meeting				x					x			
**Monitoring and evaluation**												
LF sentinel/spot check site survey					x							
**Drug logistics**												
Drug importation					x						x	
Drug transportation	x	x					x	x				
Drug storage	x	x				x	x	x				x
**Social mobilization**												
Development of IEC materials					x	x						
Dissemination of IEC materials and messages	x	x				x	x	x				x
**PC training**												
Training of trainers	x						x					
Training of supervisors						x	x				x	x
Training: CDDs	x	x					x	x				
Training: promoters	x					x	x					x
**PC preparation**												
PC preparation	x	x					x	x				
**PC drug distribution**												
LF and STH (Northern Departments)	x	x	x									
STH only		x										
LF and STH (Port-au-Prince)				x								
LF and STH (Southern Departments)							x	x	x			
**Medication for side-effects**												
Procurement of SAE drugs					x							
**Department supervision**												
Supervision	x	x	x				x	x	x			

* CDD = community drug distributor; IEC = information, education, and communication; PC = preventive chemotherapy; SAE = serious adverse effects.

### Objective 5: Facilitate the identification of integration opportunities and annual planning of NTD programs

Given that the desired number of PC treatments administered is fixed, program managers were able to determine the costs of alternative PC approaches (e.g., stand-alone or integrated NTD activities in co-endemic regions) and revise policies according to specific cost drivers. The implementation of the TIPAC in Nepal, alongside other strategic planning discussions, highlighted potential integration opportunities across the LF, STH, and trachoma programs. For example, the national NTD program in Nepal is now integrating information, education, and communication (IEC) materials for the three diseases. The program also considered integrating training procedures. However, due to the disease-specific training strategies and reports of serious adverse effects (SAEs) from previous LF PC rounds, the national program decided not to integrate training. Nepal's experience highlights the usefulness of the TIPAC for identifying integration opportunities, and it emphasizes the need to make country-specific decisions on which activities are feasible and appropriate to integrate.

## Discussion

Health systems worldwide operate under relentless fiscal pressure to curb escalating costs. The emphasis on cost containment is heightened in low- and middle-income countries where scarce resources necessitate improved resource allocation for NTD programs. Previous studies calculating the costs of implementing NTD activities have mostly consisted of retrospective analyses of PC costs [20]–[22]. The data have usually been collected from interviews or financial expenditure records and entered into spreadsheets or databases. Before the TIPAC was developed, the WHO Regional Office for Africa (AFRO) introduced a tool to support African countries in defining comprehensive budget plans in accordance with their NTD master plans. The tool allowed users to approximate the costs of a multiyear NTD program using activity-based costing and served as a precursor to the TIPAC in understanding how national programs apply a comprehensive multiyear financing tool [23]. This knowledge, combined with the recognition of the need for an annual work-planning tool to produce an accurate and detailed estimate of funding and drug requirements, guided the development of the TIPAC. The main advantages and limitations of the TIPAC and the tool implementation process are presented in Box 1.

**Box 1. Advantages and Limitations of the TIPAC and the Tool Implementation Process**

**Advantages**

- Collaborative stakeholder engagement: improves accountability and coordination; encourages programs to set realistic objectives.
- Comprehensive data collection: tool use promotes the collection of programmatic, epidemiologic, and financial data; these data can also be used by countries for other purposes (e.g., national budgeting or research).
- Itemized costing: enhances program transparency, encourages activity integration, and helps inform funding requests.
- Long-term outlook: multiyear planning improves the accuracy and validity of the data and facilitates the identification of long-term cost efficiencies.
- Tool versatility: the data entry process is flexible, and most inputs can be adjusted based on the needs of individual programs, including those targeting non-PC NTDs; a generalized version of the tool is also available for use by other programs (e.g., indoor residual spraying for malaria control).

**Limitations**

- Inter-user variability: the quality of the results depends on the accuracy and integrity of the inputs; in-country assistance from a trained facilitator is recommended to introduce the TIPAC to program managers and to provide technical support during the initial implementation round.
- Prospective planning exercise: may adversely affect the validity of data if a user believes that entering higher costs will increase funding; it is important to involve the national personnel responsible for finances and other stakeholders when estimating costs.
- Time-intensive: ten working days were usually needed to populate the TIPAC; however, the duration of the data entry process is shortened once users become accustomed to the tool and are able to use stored information from previous years.

### Generating accurate cost estimates

The ability to itemize activity costs in the TIPAC-enabled countries to generate detailed estimates of annual program costs. These cost estimates were used to inform funding requests and were helpful for countries when discussing funding strategies with stakeholders. However, like other costing tools, the TIPAC allows for some inter-user variability; the quality of the results depends on the accuracy and integrity of the inputs. As previously noted, Tanzania only provided data for 25 districts that were targeted with USAID funding, which suggests a narrow application of the TIPAC to plan and cost NTD control and elimination activities. The inclusion or exclusion of activities on the basis of the funding source biases results and represents a salient challenge when coordinating activities across multiple implementing organizations.

It is also important to remember that the costs entered into the TIPAC reflect planned expenditures and not necessarily realized expenditures. In some cases, reliance on the tool may therefore establish expectations for funding. This may impact the validity of data if a user believes that entering a cost will increase the likelihood of future funding. To minimize the inflation of the budgeted costs, it is suggested that programs start planning based on program strategies from previous years and involve the national personnel responsible for finances. Additionally, a well-trained point person in the MOH who is in charge of managing the implementation process can help to set realistic goals and cost thresholds. In Nepal, data were entered by representatives with complementary technical, programmatic, and financial expertise. Overall, a participatory data entry process that involves multiple stakeholders improves the transparency and quality of the TIPAC results.

### Calculating funding commitments and gaps

The results of the TIPAC highlighted the commitments made by governments and implementing partners. In particular, the TIPAC's automated calculation of government salaries outlined national ownership of NTD programs. Countries with limited resources may not be able to allocate substantial funds through their national budgeting process, but can show their commitment through the employment of full- and part-time employees for NTD control and elimination. Goldman and colleagues noted the importance of capturing government choices of resource allocation but faced challenges estimating the proportion of staff time spent on LF-specific efforts [21]. By allocating the entire salaries of full-time staff to program costs and apportioning the salaries of part-time staff based on the number of event and travel days entered, countries that used the TIPAC eliminated recall bias that may arise when government employees are asked to track their time spent on NTD activities.

Inter-country differences in cost allocations were expected due to variations in population sizes, disease burdens, relative purchasing power parities, levels of integration of sub-activities, and year-to-year fluctuations in program foci (e.g., changes in mapping needs and treatment histories). This study did not analyze differences in costs across countries. As costs were entered in current dollars for a given year, comparisons over time and across countries regarding funding commitments and achievement towards closing gaps would need to be converted to a base year. It would be useful, however, to monitor the progress of programs towards narrowing funding gaps over time. Levels of funding support are expected to change as donors re-evaluate funding commitments and strive to eliminate funding gaps.

### Improving coordination

For nationally-owned programs, it is important that ministries of health have a comprehensive overview of the activities that are implemented and the funding that is allocated to NTD control and elimination. This is necessary to ensure effective coordination between stakeholders and rational allocation of resources. For example, the Global NGO Deworming Inventory has noted that although many NGOs administer deworming treatments, the national programs and the wider deworming community are often unaware of these efforts [24]. By providing a structured process for sharing planned activities and funding commitments as part of annual work planning, TIPAC implementation helped to improve coordination of activities among the countries studied and discouraged the duplication of tasks. In Haiti, sharing preliminary results from the TIPAC stimulated additional partners to contribute information about their NTD prevention efforts. The process of sharing activity and cost information through the tool provided a mechanism for external partners and the MSPP to coordinate activities as part of the national strategic plan for NTDs.

During the implementation of the TIPAC, it was observed that the process can be time-consuming. If the involved personnel are not able to participate throughout the entire data entry period, it may decrease the accuracy of the cost estimates and limit the use of the tool for coordination. It is therefore recommended that countries accumulate the relevant data (e.g., target populations and unit costs) prior to implementing the TIPAC. Also, after the first year's entry, users are able to populate the tool more quickly as they become accustomed to the tool and can use the stored information from previous years. The NTD control program members in all four countries expressed positive experiences collaborating with colleagues during the data entry process; this finding may be applicable to any process or tool that helps organize and share programmatic, epidemiologic, and financial data in a transparent way.

### Facilitating integration

The integration of program elements provides an important method to reduce the transaction and administrative costs of organizing program activities and sub-activities. The similarity of the strategic approaches for the five NTDs targeted through PC delivery and the epidemiologic overlap among affected populations generate significant integration opportunities [2]. Greater program efficiencies are expected as individual disease programs integrate and reach 100% geographic coverage. As NTD programs progress, they are frequently able to improve coordination and practices, limit wasteful use of resources, and enable staff specialisation, among other cost advantages [9]. As previously mentioned, the TIPAC provided a useful platform for countries to identify integration opportunities and outline mid- to long-term program strategies.

While these efficiencies may lead to some cost savings, a consistent decrease in costs over time is not always expected for certain activities. For example, turnover rates among government staff and volunteers will generate a continuous need to retrain personnel. Programs also experience diseconomies of scale and scope when targeting hard-to-reach populations. In addition, costs may increase towards the end of a program's lifespan, when impact assessments are implemented to monitor the effectiveness of interventions and to measure progress towards control and elimination goals. It is therefore important to not rely exclusively on financing estimates based on average costs that assume that costs will monotonically decrease over time.

### Conclusions

Implementing the TIPAC in these four countries offered an opportunity to assess the feasibility of using a versatile costing instrument to inform and facilitate resource planning. In the study countries, the TIPAC provided results that were concordant with the objectives of tool use; the implementation process also helped identify key lessons to improve future use. Populating the tool promoted synergistic efforts between national NTD programs and partners to estimate costs accurately, coordinate activities, identify integration opportunities, and achieve program goals to control and eliminate targeted diseases. Although the TIPAC is not a substitute for the strategic process of developing a national plan of action, it should strongly align with this document to improve resource planning. Once the financial landscape is evaluated through the TIPAC, a program manager can better decide the rate at which scale-up is possible, what activities should be postponed until funding is available, and the quantity of drugs needed to support program activities.

Since the development of the TIPAC for national NTD control programs, the tool has also been modified and generalized for wider applicability. It has been adapted as a platform to cost and plan malaria control activities (e.g., indoor residual spraying [IRS]) and HIV programs. Additionally, a generic version of the tool is being developed that can be applied to other health interventions. The impetus to apply the TIPAC process to other projects is due to the growing need for national programs and donors to demonstrate efficiencies and to collaborate when scaling-up activities and forecasting future needs.

The TIPAC was recently recognized by WHO as a recommended planning and costing tool for national NTD programs [25]. It can be used on an annual basis for PC drug applications to populate the WHO Joint Request for Selected PC Medicines. Partners are also working with WHO, WHO-AFRO, and other regions to introduce capacity building workshops for the tool. Overall, the implementation of the TIPAC in multiple countries provided a unique opportunity to assess the challenges of applying a costing instrument for country programs. The data and lessons gathered from this experience will help standardize NTD control and elimination planning.

## Supporting Information

Table S1 Default tool PC activities and subactivities.
(DOC)

Text S1 PC target population and drug demand algorithms.
(DOC)
