# Supplementary material for: Resource Planning for Neglected Tropical Disease (NTD) Control Programs: Feasibility Study of the Tool for Integrated Planning and Costing (TIPAC)
Source: PLoS Negl Trop Dis. 2014 Feb 27;8(2):e2619. doi: 10.1371/journal.pntd.0002619 (PMC3937228; doi:10.1371/journal.pntd.0002619)
Supplement: Table S1 — Default tool PC activities and subactivities. (DOC) [file pntd.0002619.s001.doc]

**SUPPORTING INFORMATION LEGENDS**

**Table S1.** Default tool PC activities and sub-activities.

| **Activity** | **Sub-activity** | **Definition** |
| --- | --- | --- |
| **Strategic Planning** | Annual review meeting | Review the status of the national NTD program and long-term goals. |
|  | National stakeholders meeting | Coordinate efforts between key stakeholders. |
|  | Technical planning meeting | Discuss current guidelines and logistic aspects of the control and elimination of NTDs; development and planning of specific sub-activities when appropriate. |
| **Advocacy** | National advocacy | Encourage greater national government participation and support for NTD control and elimination. |
|  | Regional/district advocacy | Encourage greater regional and district government participation and support for NTD control and elimination. |
|  |  |  |
| **Mapping** | Integrated Mapping | Capture prevalence rates for multiple diseases using a single collection tool and survey platform (i.e. integrated approach); carried out in accordance with guidelines recommended by WHO and its partners. |
|  | LF, Onchocerciasis, SCH, STH, and Trachoma Mapping | Evaluate district disease landscapes and prevalence (i.e. disease-specific mapping); carried out in accordance with guidelines recommended by WHO and its partners. |
|  |  |  |
| **Monitoring and Evaluation (M&E)** | LF sentinel/spot check site survey | Collect and analyze LF prevalence data from designated geographic locations for monitoring and evaluation. |
|  | LF transmission assessment survey | Assess whether LF prevalence rates have decreased below a threshold level to determine if MDA should be discontinued; the survey is also conducted during post-MDA surveillance data collection. |
|  | Onchocerciasis entomological survey | Determine the abundance of onchocerciasis disease vectors. |
|  | Onchocerciasis epidemiological survey | Assess population onchocerciasis microfilariae levels. |
|  | SCH and/or STH prevalence survey/sentinel sites | Collect and analyze schistosomiasis and STH prevalence data from designated geographic locations for monitoring and evaluation. |
|  | PC coverage survey | Validate reported PC coverage. |
|  | Serious adverse effects (SAE) monitoring | Observe and treat individuals suffering serious adverse reactions from PC. |
|  | Trachoma prevalence study | Population-based impact assessment to determine prevalence of trachoma in a designated administrative unit. |
|  |  |  |
| **Morbidity Control and Surgery** | Hydrocele surgery, lymphodema management, and trichiasis surgery | Implement measures to manage morbidity and prevent further disability. |
|  |  |  |
| **Vector Control** | Mosquito, black fly, and snail control | Implement strategies to reduce or interrupt disease transmission by controlling vectors. |
|  |  |  |
| **Drug Logistics** | Drug transportation | Transport drugs to regional and district storage facilities and communities. |
|  | Drug importation | Ship and clear donated and/or procured drugs. |
|  | Drug repackaging | Repackage drugs into suitable doses and regimens. |
|  | Drug storage | Manage and store drug stock. |
|  |  |  |
| **Social Mobilization** | Development of IEC materials | Develop materials for social mobilization campaigns. |
|  | Dissemination of IEC materials and messages | Sensitize local communities to improve community awareness and health education. |
|  |  |  |
| **Training** | Development/printing of training materials | Develop materials for training of participants. |
|  | Training of trainers | Instruct health workers responsible for the administration and functionality of PC activities. |
|  | Training of supervisors | Instruct health workers responsible for the supervision of PC activities. |
|  | Training: Teacher/health worker (school-based PC delivery) | Instruct teachers and health workers responsible for the implementation of PC activities in schools. |
|  | Training: CDDs (community-based PC delivery) | Instruct community health workers and volunteers responsible for the implementation of PC activities in communities. |
|  | Refresher training: Teacher/health worker (school-based PC delivery) | Re-instruct teachers and health workers responsible for the implementation of PC activities in schools. |
|  | Refresher training: CDDs (community-based PC delivery) | Re-instruct community health workers and volunteers responsible for the implementation of PC activities in communities. |
|  |  |  |
| **PC Registration** | Registration (census) | Gather accurate information on the size and eligibility of the target populations. |
|  |  |  |
| **PC Drug Distribution** | Drug distribution | Manage and administer PC drugs. |
