# Supplementary material for: Resource Planning for Neglected Tropical Disease (NTD) Control Programs: Feasibility Study of the Tool for Integrated Planning and Costing (TIPAC)
Source: PLoS Negl Trop Dis. 2014 Feb 27;8(2):e2619. doi: 10.1371/journal.pntd.0002619 (PMC3937228; doi:10.1371/journal.pntd.0002619)
Supplement: Text S1 — PC target population and drug demand algorithms. (DOC) [file pntd.0002619.s002.doc]

**SUPPORTING INFORMATION LEGENDS**

**Text S1.** PC target population and drug demand algorithms.

The TIPAC automatically estimates PC target populations and drug requirements based on population data and district selections. The target population formula accounts for multiple treatment rounds while avoiding potential double counting of individuals targeted for more than one disease by the same drug regimen. Once the PC target population for each NTD is calculated, a multiplier for the associated medicine is applied to this target population to determine drug needs in the PC Drug Acquisition module. When calculating required drug quantities, the TIPAC allows users to specify: (i) which of two different drug multipliers to apply depending on the schistosomiasis age group targeted (i.e. school age children or high-risk adults) and (ii) whether treatment for LF and STH is integrated to determine the demand for albendazole.

These formulas internalize certain assumptions. First, for onchocerciasis or STH with two rounds of treatment, the calculations assume that those treated in the second round also received treatment in the first round. Second, the tool assumes maximum integration of drug packages for LF and onchocerciasis PC delivery; therefore, in LF-endemic districts that are co-endemic for onchocerciasis, at least one round of ivermectin per year is assumed to cover both the LF and onchocerciasis target populations.
